# Cell-to-cell spread inhibiting antibodies constitute a correlate of protection against herpes simplex virus type 1 reactivations: A retrospective study

**DOI:** 10.3389/fimmu.2023.1143870

**Published:** 2023-03-16

**Authors:** Mira Alt, Susanne Wolf, Lukas van de Sand, Robin Dittrich, Tobias Tertel, Leonie Brochhagen, Miriam Dirks, Ulrich Wilhelm Aufderhorst, Laura Thümmler, Mona Otte, Kordula Rainer, Ulf Dittmer, Bernd Giebel, Mirko Trilling, Christiane Silke Heilingloh, Ramin Lotfi, Michael Roggendorf, Oliver Witzke, Adalbert Krawczyk

**Affiliations:** ^1^ Institute for Virology, University Hospital Essen, University of Duisburg-Essen, Essen, Germany; ^2^ Department of Infectious Diseases, University Hospital Essen, University of Duisburg-Essen, Essen, Germany; ^3^ Institute for Transfusion Medicine, University Hospital Essen, University of Duisburg-Essen, Essen, Germany; ^4^ Institute for Clinical Transfusion Medicine and Immunogenetics Ulm, German Red Cross Blood Transfusion Service Baden-Württemberg - Hessen and University Hospital Ulm, Ulm, Germany; ^5^ Institute for Transfusion Medicine, University of Ulm, Ulm, Germany

**Keywords:** HSV-1, cell-to-cell spread, antibodies, reactivation, protection

## Abstract

**Background:**

Herpes simplex viruses (HSV) cause ubiquitous human infections. For vaccine development, knowledge concerning correlates of protection is essential. Therefore, we investigated (I) if humans are in principle capable producing cell-to-cell spread inhibiting antibodies against HSV and (II) whether this capacity is associated with a reduced HSV-1 reactivation risk.

**Methods:**

We established a high-throughput HSV-1-ΔgE-GFP reporter virus-based assay and evaluated 2,496 human plasma samples for HSV-1 glycoprotein E (gE) independent cell-to-cell spread inhibiting antibodies. Subsequently, we conducted a retrospective survey among the blood donors to analyze the correlation between the presence of cell-to-cell spread inhibiting antibodies in plasma and the frequency of HSV reactivations.

**Results:**

In total, 128 of the 2,496 blood donors (5.1%) exhibited high levels of HSV-1 gE independent cell-to-cell spread inhibiting antibodies in the plasma. None of the 147 HSV-1 seronegative plasmas exhibited partial or complete cell-to-cell spread inhibition, demonstrating the specificity of our assay. Individuals with cell-to-cell spread inhibiting antibodies showed a significantly lower frequency of HSV reactivations compared to subjects without sufficient levels of such antibodies.

**Conclusion:**

This study contains two important findings: (I) upon natural HSV infection, some humans produce cell-to-cell spread inhibiting antibodies and (II) such antibodies correlate with protection against recurrent HSV-1. Moreover, these elite neutralizers may provide promising material for immunoglobulin therapy and information for the design of a protective vaccine against HSV-1.

## 1 Introduction

Herpes simplex viruses (HSV) types 1 and 2 are among the most common human infections. Globally, more than 3.7 billion people are infected with HSV-1 (1) and nearly 500 million with HSV-2 (2). Both viruses cause a broad range of disease manifestations ranging from painful and irritating but self-limiting oral or genital lesions to severe disseminated and life-threatening infections in immunocompromised patients (2–5). Serious complications can also be observed in patients suffering from ocular herpes infections, which may result in irreversible damage of the eye or even blindness (6, 7).

Until today, an approved vaccine is not available (8). Numerous animal studies investigating the efficacy of distinct vaccine candidates such as inactivated virus particles, live- or genetically attenuated viruses or recombinant subunit vaccines yielded promising results (9, 10). However, none of the vaccine candidates being tested in clinical trials have been effective (8). The GlaxoSmithKline (GSK) Herpevac trial using a recombinant HSV-2 glycoprotein D (gD2) subunit vaccine was largest clinical trial performed so far (11). However, the vaccine showed some protection against HSV-1 (11). The discrepancy between promising results of animal studies and the failure of clinical trials in humans suggest a fundamental difference in the immune response to HSV in mice or guinea pigs and humans. A retrospective study uncovered differences in antibody responses between humans and rodents concerning virus-specific antibodies, neutralizing antibodies, and cell-to-cell spread inhibiting, neutralizing antibodies (CCSi-NAbs). Most recently, the antibody responses to the gD2 subunit vaccine were analyzed in humans and guinea pigs (12). Antibodies produced by vaccinated humans recognized significantly fewer crucial gD2 epitopes as compared to guinea pig antibodies (12, 13). The crucial gD2 epitopes are targets of neutralizing or cell-to-cell spread inhibiting antibodies (14). The cell-to-cell spread of HSV is known as a mechanism of immune evasion, and markedly facilitates the spread of HSV upon reactivation (14). Antibodies, which can block this route of viral transmission are associated with protection from disease in mouse models (12, 15). Previously, we developed a highly neutralizing and cell-to-cell spread inhibiting monoclonal antibody (mAb) called 2c. This antibody mediates almost complete protection from lethal genital HSV-1 infection - even in highly immunodeficient NOD/SCID mice (15, 16). Moreover, mAb 2c protects mice from the development of severe ocular infections (17–19). Importantly, mAb 2c is significantly more effective in protecting from disease than polyclonal human neutralizing antibodies used at a similar neutralizing titer, highlighting the importance of the inhibition of cell-to-cell spread in protecting from disease (20). These *in vitro* and *in vivo* data demonstrated that neutralizing antibodies, which inhibit the cell-to-cell spread are superior to antibodies that “just” neutralize but do not inhibit the cell-to-cell spread (20). These findings raise the apparent question, if the inhibition of the cell-to-cell spread might contribute to protection from primary and/or recurrent disease. Intriguingly, the re-evaluation of the GSK Herpevac trial revealed that gD2-immunized individuals only barely produced antibodies that targeted gD2 epitopes associated with cell-to-cell spread (13), raising the fundamental question whether humans are in principle able to produce cell-to-cell spread inhibiting antibodies against HSV.

To address this question, we established a HSV-1 GFP reporter virus-based high-throughput screening assay, tested 2,496 plasma samples for cell-to-cell spread inhibiting antibodies, and verified these results using wild type HSV. We show for the first time that a small proportion of humans (“elite responders”) indeed produced functional amounts of cell-to-cell spread inhibiting antibodies and - even more striking - that above concentrations such antibodies correlatively protect from HSV reactivation. The obtained data, imply cell-to-cell spread inhibiting antibodies might provide a promising tool in treating severe HSV-1 infections.

## 2 Methods

### 2.1 Sera and plasma

Sera and plasma samples were harvested during routine blood donations at the Institute of Transfusion Medicine, University of Ulm, Germany. For the initial high-throughput screening for cell-to-cell spread inhibiting plasmas with the HSV-1-ΔgE-GFP reporter virus, 2,643 plasmas from healthy blood donors between the age of 18 and 65 were randomly selected. Of these, 147 (5.6%) were tested HSV-1 negative and 2,496 (94.4%) HSV-1 positive. Written informed consent was obtained from all participants.

### 2.2 Viruses

HSV-1 strain F and HSV-1-ΔgE-GFP reporter virus were propagated in Vero cells and stored at −80°C. Both viruses were kindly provided by Hartmut Hengel (Institute of Virology, Freiburg, Germany). HSV-1-ΔgE-GFP was initially described by Farnsworth et al. (21). Viral titers were determined by a standard endpoint dilution assay and calculated as 50% tissue culture infectious dose (TCID_50_)/ml as previously described (22).

### 2.3 Cells

Vero cells (American Type Culture Collection, ATCC, CCL81, Rockville, MD) were cultured in Dulbecco’s Modified Eagle Medium (DMEM, Life Technologies Gibco, Darmstadt, Germany) containing 10% (v/v) fetal calf serum (FCS; Life Technologies Gibco), 100 U/ml penicillin and 0.1 mg/ml streptomycin.

### 2.4 Antibodies

Monoclonal antibodies mAb hu2c and mAb 2c were purified from serum-free SP2/0- or hybridoma cell supernatants by chromatography with protein A agarose according to the manufacturer’s protocol (Thermo Fisher Scientific, Waltham, MA, USA) and as described previously (15, 16). Labeling of the antibody 2c was performed with the Alexa Flour™ 488 Protein Labeling Kit (Thermo Fisher Scientific, Waltham, MA, USA) according to the manufacturer’s protocol. Concentration was measured with a NanoDrop 2000 spectrometer (Thermo Fisher Scientific, Waltham, MA, USA).

### 2.5 HSV-1-ΔgE-GFP based screening for cell-to-cell spread inhibiting antibodies

To investigate whether humans are able to produce cell-to-cell spread inhibiting antibodies, we established an HSV-1-ΔgE-GFP reporter virus-based assay for the high-throughput screening of HSV-1 seropositive human serum or plasma samples. The assay was evaluated using the HSV-1 cell-to-cell spread inhibiting antibody mAb hu2c (15). Alphaherpesviruses lacking gE were described as replication competent but showed a reduced capacity for cell-to-cell spread (23). Thus, we decided to use HSV-1-ΔgE-GFP for the initial screening of donor plasmas for cell-to-cell spread inhibiting antibodies. Due to a reduced capacity for cell-to-cell spread of HSV-1-ΔgE-GFP, we expected to detect even low levels of cell-to-cell spread inhibiting antibodies from human plasma.

Highly permissive Vero cells were seeded on 24-well plates at a density of 1 x 10^5^ cells/well. Confluent cell cultures were infected with 200 TCID_50_ HSV-1-ΔgE-GFP/well (MOI = 0.001). After two hours of incubation, the inoculation medium was removed and the cell cultures were incubated with serial dilutions of mAb hu2c (0 – 500 nM). Commercial polyclonal human antibody preparations, Cytotect and Intratect (Biotest, Dreieich, Germany), were used as controls at a concentration of 1 or 2 mg/ml. These intravenous immunoglobulins are a type of medication made from the blood plasma of several thousand donors. They are primarily composed of IgG antibodies, which are polyclonal and polyvalent due to the manufacturing process. They also contain small amounts of IgA, IgE, and IgM. To standardize the background levels, all purified antibodies were applied in medium containing serum or plasma from an HSV-1 and HSV-2 seronegative donor at a 1:40 (v/v) dilution. With the plasma concentration used, it can be assumed that cell-free spreading does not occur due to the high concentration of neutralizing antibodies. If the concentration of neutralizing antibodies in the corresponding plasma sample is very low, spreading *via* the supernatant is accepted for screening. After three days of incubation, the plaque formation was examined by fluorescence microscopy (Axio Observer D1, Zeiss). Additionally, the fluorescence levels were quantified. For this purpose, the cell culture medium was removed, the cells washed with PBS, detached with Trypsin/0.5% EDTA (Life Technologies Gibco), resuspended and transferred to 96-well plates. GFP-signals were quantified using the Mithras² LB 943 microplate multimode reader (Berthold Technologies, Bad Wildbad, Germany) (24).

### 2.6 High throughput screening of plasmas for the inhibition of HSV-1 cell-to-cell spread

A total number of 2,496 human plasmas were screened for the inhibition of the HSV-1 cell-to-cell spread using the high throughput assay as described above. Human plasma samples were applied at 1:40 dilutions. The monoclonal humanized antibody mAb hu2c served as positive control at a concentration of 500 nM (75 µg/ml) diluted in plasma from an HSV-1/2 seronegative donor (1:40 in cell culture medium). At this concentration of mAb hu2c, the HSV-1 cell-to-cell spread is completely blocked. After 72 hours of incubation, the GFP-signal was measured using the Mithras² LB 943 microplate multimode reader (Berthold Technologies). Fluorescence-values for individual plasma samples were compared with the GFP-intensity measured for mAb hu2c for each plate. The values obtained for the plasma samples were then normalized to the mAb hu2c control and calculated as the x-fold value of the mAb hu2c signal.

### 2.7 Identification and quantification of HSV-seropositive plasmas by ELISA

The HSV-seropositivity status of donors completing the survey was confirmed by ELISA using the anti-herpes simplex virus type 1 and 2 IgG human ELISA kit (abcam, Cambridge, United Kingdom). The binding HSV-1 IgG ELISA antibody titers of the blood donors taking part in the retrospective survey were quantified with the anti-herpes simplex virus type 1 (gC1) IgG ELISA kit (Euroimmun, Lübeck, Germany). Both tests were performed according to manufacturer’s instructions.

### 2.8 Retrospective survey to determine the frequency of HSV-reactivations and general data

To investigate the role of HSV-1 cell-to-cell spread inhibiting antibodies in HSV-seropositive people, we assessed the frequency of symptomatic HSV reactivations in the frame of a retrospective survey. For this purpose, we invited 128 “elite donors” as well as an equal number of volunteers from the “partial-inhibition” and “no-inhibition” group to participate the retrospective survey and to donate additional plasma and serum for subsequent analysis. The response rate ranged from 41% to 47%, depending on the group. The data acquisition comprised the annual numbers of symptomatic oral or genital HSV-reactivations characterized by the occurrence of characteristic lesions. Furthermore, data on general topics like age, gender, body mass index (BMI) as well as smoking behavior were collected. The survey enrolled a total number of 158 blood donors stratified in three comparable groups according to the presence of cell-to-cell spread inhibiting antibodies as complete inhibition (n = 47), partial inhibition (n = 58) and no inhibition (n = 53).

### 2.9 Neutralization assay

To investigate the neutralizing antibody titers in blood donor plasmas, a neutralization assay was performed as previously described (25). Briefly, serial dilutions (1:20 to 1:2560) of the respective plasma samples were pre-incubated with 100 TCID_50_ of HSV-1 F for one hour at 37°C and added afterwards to confluent Vero cells cultured in 96-well microtiter plates. After 72 hours, the cytopathic effect was analyzed by microscopy and the reciprocal neutralization titer was determined.

### 2.10 Flow cytometry analysis of HSV-1 wild type virus cell-to-cell spread inhibition

Initially, “elite donors” whose plasmas contain cell-to-cell spread inhibiting antibodies were identified using the high throughput HSV-1 ΔgE GFP reporter virus-based assay. To investigate whether these plasmas are capable of inhibiting the cell-to-cell spread of a HSV-1 wild type virus, we established a flow cytometry-based assay with HSV-1 F. Confluent Vero E6 cells grown in 24-well plates were infected with 1000 TCID_50_/mL of HSV-1 F for two hours at 37°C and 5% CO_2_. Subsequently, the inoculation medium was removed and medium containing plasma samples at a 1:40 (v/v) dilution were incubated for two days. Due to the sufficiently high concentration of neutralizing antibodies in the selected plasmas, it can be assumed that cell-free spreading no longer occurs. As positive control, we used mAb hu2c as described above. Cells were harvested and washed with 1% (v/v) FSC/PBS, stained with the monoclonal antibody mAb 2c-AF488 and analyzed by BD FACSAria IIIu (BD Bioscience, Franklin Lakes, NJ, USA). The cell count measured for the plasmas were normalized to the mAb hu2c control (500 nM).

### 2.11 Statistical analysis

Statistical analysis was performed using GraphPad Prism 9 (San Diego, CA, USA). *In vitro* data were statistically analyzed using a one-way analysis of variance (ANOVA) test followed by the Dunn’s multiple comparison *post-hoc* test. Correlation of data was performed by Spearman’s rank correlation test. Study cohort calculations were analyzed by Fischer´s exact test and Two-way ANOVA. Data describe biological replicates.

### 2.12 Study approval

The study was performed in accordance with The Code of Ethics of the World Medical Association (Declaration of Helsinki) and was approved by the ethical committee of the University of Ulm and the University Hospital Essen (15-6360-BO).

## 3 Results

To evaluate whether humans are able to produce potent spreading inhibiting anti-HSV antibodies upon natural HSV-1 infection, we established a high-throughput assay to test human plasma and serum samples for their impact on HSV-1 cell-to-cell spread.

### 3.1 HSV-1-ΔgE-GFP reporter virus-based screening assay for cell-to-cell spread inhibiting antibodies

**Figure 1:**
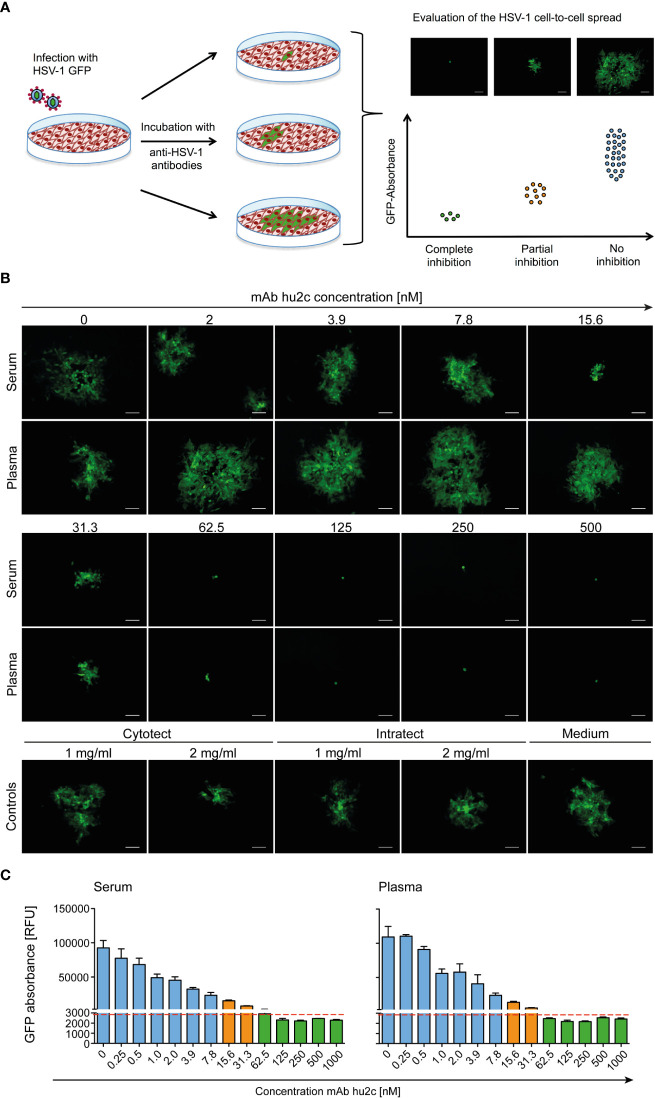
Assessment of the HSV-1 cell-to-cell spread inhibiting antibodies of human plasma, serum samples or antibodies using the HSV-1 ΔgE GFP reporter virus-based screening method. **(A)** The procedure was based on assessing the extent of plaque formation, which was proportional to the GFP-signal emitted by the infected cells. Confluent Vero cells were infected with HSV-1 ΔgE GFP reporter virus at low MOI. Infected cell cultures were overlaid with a medium containing either sera or plasma samples from HSV-seropositive humans at a 1:40 dilution. After 72 h hours of incubation, plaque formation was qualitatively assessed by fluorescence microscopy and simultaneously the GFP-signal was quantified as relative fluorescence units (RFU). **(B)** The performance of the HSV-1 ΔgE GFP reporter virus-based assay was evaluated for the screening of sera and plasma that contain various concentrations of HSV-1 cell-to-cell spread inhibiting antibodies. Confluent Vero cells growing on 24-well plates were infected with 200 TCID50/500 µl of the HSV-1 ΔgE GFP reporter virus. After 2 h of incubation, the inoculating medium was removed and the cells were overlaid with a medium containing sera or plasma from a HSV-1 seronegative donor at a 1:40 dilution. Additionally, the monoclonal, HSV-1/2 cell-to-cell spread inhibiting antibody mAb hu2c was added at a final concentration ranging from 0 to 1000 nM. After 72 h hours, plaque formation, which indicates HSV-1 spread via the cell-to-cell spread, was qualitatively assessed by fluorescence microscopy. 100x magnification, scale bar = 100 µm. **(C)** Additionally, the cell cultures were transferred to 96-well plates to quantify the GFP-signal as relative fluorescence units (RFU). Dashed line = cell-to-cell spread inhibiting concentration of mAb hu2c. Green bars = complete inhibition, orange = partial inhibition, blue = no inhibition of HSV-1 cell-to-cell spread.

The screening assay is based on the quantification of the progressing plaque expansion, which is representative for the extent HSV-1 of cell-to-cell spread. By using the HSV-1-ΔgE-GFP reporter virus, plaque formation, which is proportional to the GFP expression level, could be quantified using a fluorescence reader and visualized by fluorescence microscopy (**Figure 1A**). Confluent Vero cell monolayers were infected with the HSV-1-ΔgE-GFP reporter virus at a low multiplicity of infection (MOI = 0.001). Thereby, only very few cells within the cell layer become infected. Afterwards, medium containing the sample to be tested, e.g. serum, plasma or purified antibodies was added to the infected cell cultures (**Figure 1A**). The test was evaluated 3 days after infection in a quantitative manner by assessing the GFP signal and in a qualitative manner by fluorescence microscopy (**Figure 1A**). Single infected cells accompanied by a low GFP signal represent a complete inhibition of the cell-to-cell spread, since antibodies prevented plague formation. Unrestricted plaque expansion and strong GFP signals at levels similar to those of the HSV-1 seronegative control were scored as no inhibition of the cell-to-cell spread. Small plaques and moderate GFP signals indicated the presence of cell-to-cell spread inhibiting antibodies in the sample, even if there was no complete inhibition of the cell-to-cell spread (**Figure 1A**).

The HSV-1-ΔgE-GFP-based high-throughput screening assay was first evaluated using the humanized antibody mAb hu2c that is known to completely inhibit HSV-1 and HSV-2 cell-to-cell spread (**Figures 1B, C**). Confluent Vero cell cultures were infected with HSV-1-ΔgE-GFP and subsequently incubated with medium containing graded concentrations (0 - 500 nM) of mAb hu2c. Plasma or serum from an HSV-1 and HSV-2 double seronegative donor was added at a 1:40 dilution. Complete inhibition of the cell-to-cell spread could be observed at mAb hu2c concentrations between 125 and 500 nM (**Figure 1B**). Almost complete inhibition was observed at 62.5 nM. At this concentration, only very small plaques with a maximum of 4 infected cells/plaque were visible (**Figure 1B**) and the quantitative analysis showed an almost unchanged GFP signal compared to higher mAb hu2c concentrations (**Figure 1C**). This concentration represents the lowest mAb hu2c concentration that almost completely inhibits the cell-to-cell spread (**Figure 1B**, dashed line). In contrast, between 2 and 7.8 nM of mAb hu2c, there was no visible reduction of the cell-to-cell spread (**Figure 1B**) and the GFP signal was notably higher when compared to concentrations above 62.5 nM mAb hu2c (**Figure 1C**). Interestingly, plaques were smaller at mAb hu2c concentrations between 15.6 and 31.3 nM mAb hu2c (**Figure 1B**) accompanied by only slightly increased GFP-signals (**Figure 1C**), indicating a partial inhibition of the cell-to-cell spread at these concentrations.

These data show that the quantitative measurement of the GFP signal correlates with the plaque expansion observed in cell-culture, which obviously represents the extent of the HSV-1 cell-to-cell spread. Furthermore, our assay was able to distinguish complete, partial, and no inhibition of the HSV-1 cell-to-cell spread.

### 3.2 Identification of HSV-1 elite responders with cell-to-cell spread inhibiting antibodies

**Figure 2:**
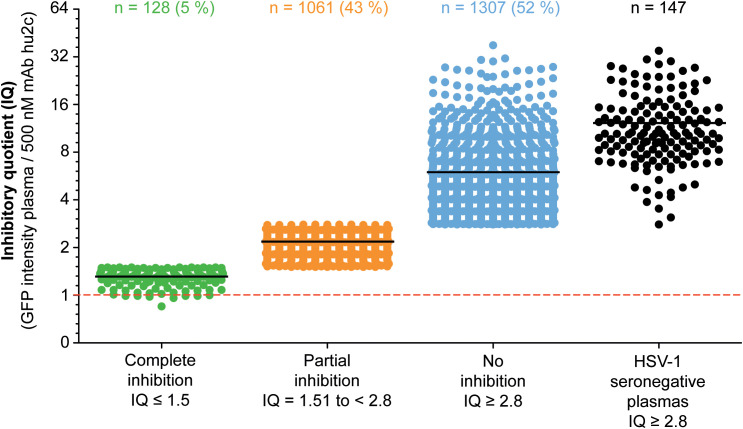
Assessment of the HSV-1 cell-to-cell spread inhibition capacity of plasma samples from HSV-1 seropositive blood donor. A total number of 2,496 plasma samples from blood donors were investigated for HSV-1 cell-to-cell spreading properties using a HSV-1 ΔgE GFP reporter virus-based assay as described above. The efficacy of the plasma samples regarding cell-to-cell spread inhibition is shown as a fold change of the 500 nM mAb hu2c threshold (dashed line). At this concentration mAb hu2c completely inhibits the HSV-1 cell-to-cell spread. The efficacy of the plasma samples regarding cell-to-cell spread inhibition was determined by dividing the GFP-signal of a cell culture treated with a plasma sample through the GFP-signal of mAb hu2c control. This quotient was termed inhibitory quotient (IQ) and represents the x-fold value of the GFP-signal measured for mAb hu2c. The plasma samples were classified as completely cell-to-cell spread inhibiting (green dots, IQ ≤ 1.5), partially inhibiting (orange dots, IQ = 1.51 - 2.8) and non-inhibiting (blue dots, IQ ≥ 2.8). Each point represents the IQ for each donor; horizontal bars represent the median value.

To investigate whether humans can produce HSV-1 cell-to-cell spread inhibiting antibodies, plasma samples from 2,496 blood donors were screened for their cell-to-cell spread inhibiting properties. All samples were tested using the high-throughput screening assay described above. A mAb hu2c positive control was included on each 24-well plate. None of the 147 HSV-1 seronegative control plasmas exhibited partial or complete cell-to-cell spread inhibition, demonstrating the specificity of the assay. The efficacy of the plasmas regarding cell-to-cell spread inhibition was determined by dividing the GFP-signal of a culture containing the plasma of interest through the GFP-signal of the mAb hu2c-treated control exhibiting “complete inhibition”. This quotient was termed inhibitory quotient (IQ) and represents the x-fold value of the GFP-signal measured for mAb hu2c. Plasmas were stratified according to their cell-to-cell spread inhibiting activity as completely inhibiting (IQ ≤ 1.5), partially inhibiting (IQ = 1.51 to < 2.8) and non-inhibiting (IQ ≥ 2.8). In total, 128 (5.1%) of the plasmas showed complete and 1,061 (42.5%) exhibited partial inhibition (**Figure 2**). The fold change of the remaining 1,307 (52.4%) plasmas was in a similar range as plasmas derived from HSV-1 seronegative donors and had no effect on the cell-to-cell spread.

### 3.3 Assessment of the frequency of HSV reactivations in plasma donors

**Figure 3:**
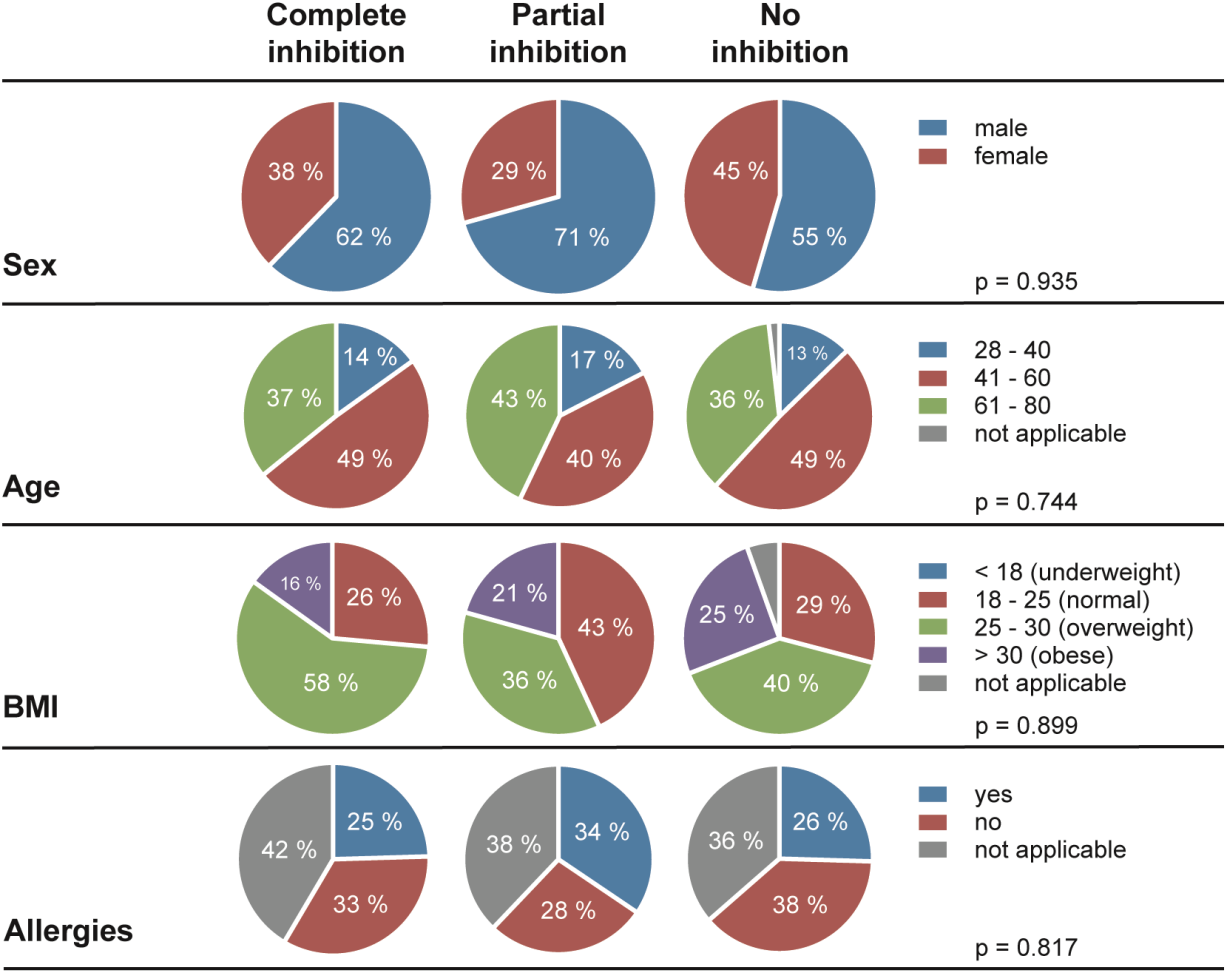
Demographic characteristics of the study cohort. In total, 158 of HSV-1 positive blood donors (complete inhibition, n = 47; partial inhibition, n = 58 and no inhibition, n = 53) were participated the retrospective survey. Differences between the three cohorts in terms of sex, age, Body-Mass-Index (BMI), and allergies were statistically evaluated using a Two-way analysis of variance test (ANOVA).

**Figure 4:**
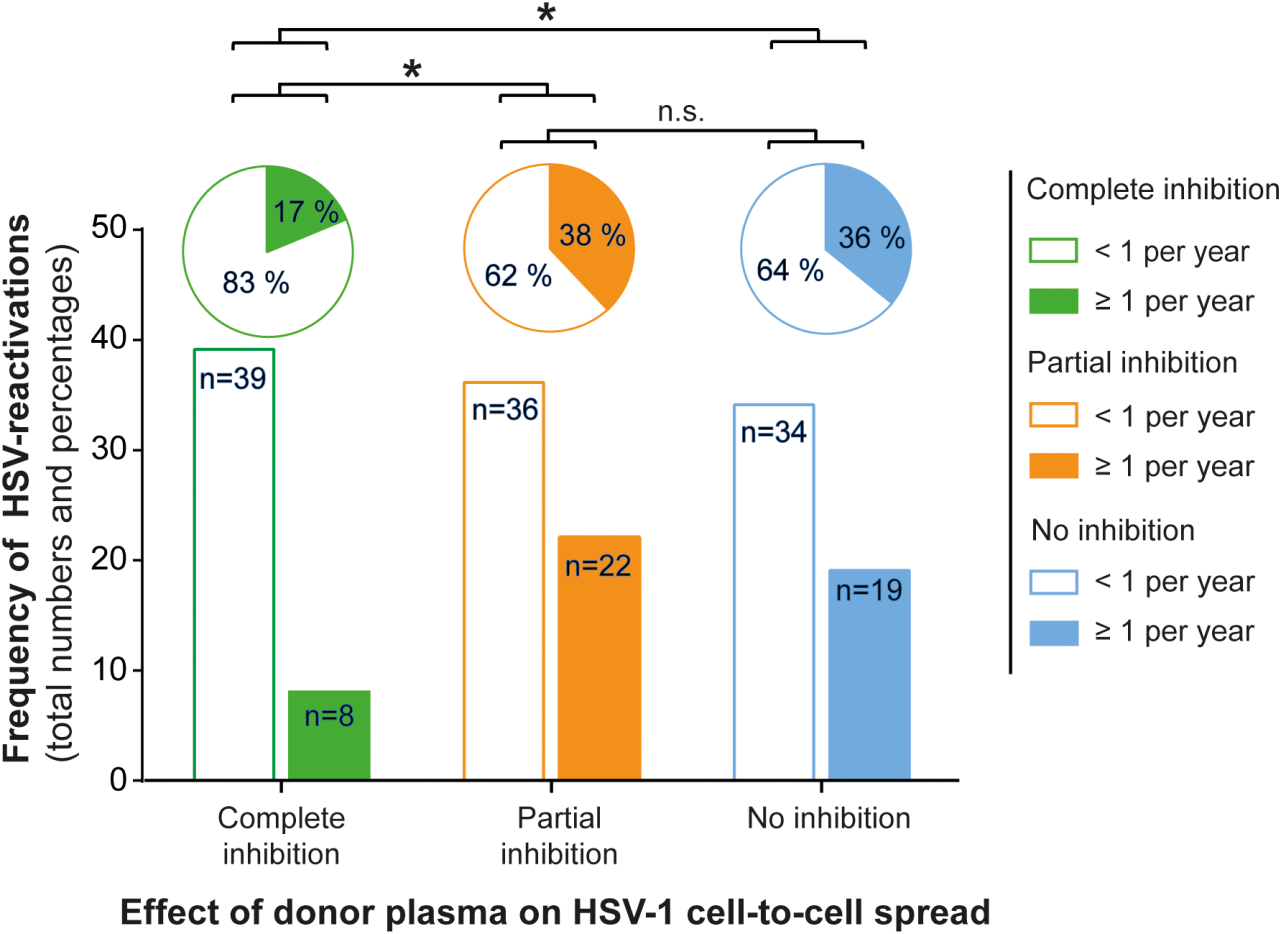
Correlation between protective antibody response and the frequency of HSV reactivation. A total number of 158 HSV seropositive blood donors previously being tested for cell-to-cell spread inhibiting antibodies were retrospectively interviewed for the frequency of symptomatic HSV reactivations per year. The donors were divided into the three groups (complete inhibition, n = 47; partial inhibition, n = 58 and no inhibition, n = 53) according to the performance of the donor plasmas on the HSV-1 cell-to-cell spread inhibition. The total numbers of donors are depicted as a bar chart and the percentages as a pie chart above. Differences in the annual frequency of HSV reactivation were analyzed using the Fischer´s exact test. Significant changes (^∗^p < 0.05) are indicated by asterisks and non-significant changes (p > 0.05) are labeled as “n.s.”.

Next, we assessed the frequency of HSV reactivations in HSV-1 positive blood donors to investigate whether there is a correlation between the presence of cell-to-cell spread inhibiting antibodies and the frequency of reported reactivations. For this purpose, we conducted a retrospective survey including 158 blood donors that were randomly selected from group with complete inhibiting antibodies (elite neutralizers; n = 47), the partial inhibition group (n = 58) and the no-inhibition group (n = 53). The HSV-seropositive status of all these donors was confirmed by HSV-1 IgG ELISA. The biometric characteristics of the three different cohorts showing either complete, partial, or no cell-to-cell spread inhibition are summarized in **Figure 3**. All three groups were comparable regarding mean age, gender, smoking behavior as well as the mean body mass index (BMI). Next, the three different groups were interrogated regarding the frequency of reported HSV reactivations. The frequency of HSV reactivations was recorded according to the observed occurrence of oral or genital lesions with less than one time per year or one or more symptomatic reactivations per year (< 1 or ≥ 1 reactivation per year). Interestingly, study participants from the elite neutralizer group showed significant lower frequencies of HSV reactivation as compared to the groups that showed only partial or no cell-to-cell spread inhibition capacity (**Figure 4**). Only 17% of individuals from the elite neutralizer group reported one or more reactivation per year, whereas the frequency of at least one reactivation per year was 38% in the partial inhibition group and 36% in the no inhibition group. These data clearly demonstrate a significant correlation between the presence of cell-to-cell spread inhibiting antibodies and a lower rate of HSV-reactivation, providing a strong argument for their functional relevance in preventing recurrent herpes disease.

### 3.4 Assessment of HSV-1 binding antibody titers by HSV-1 IgG ELISA

**Figure 5:**
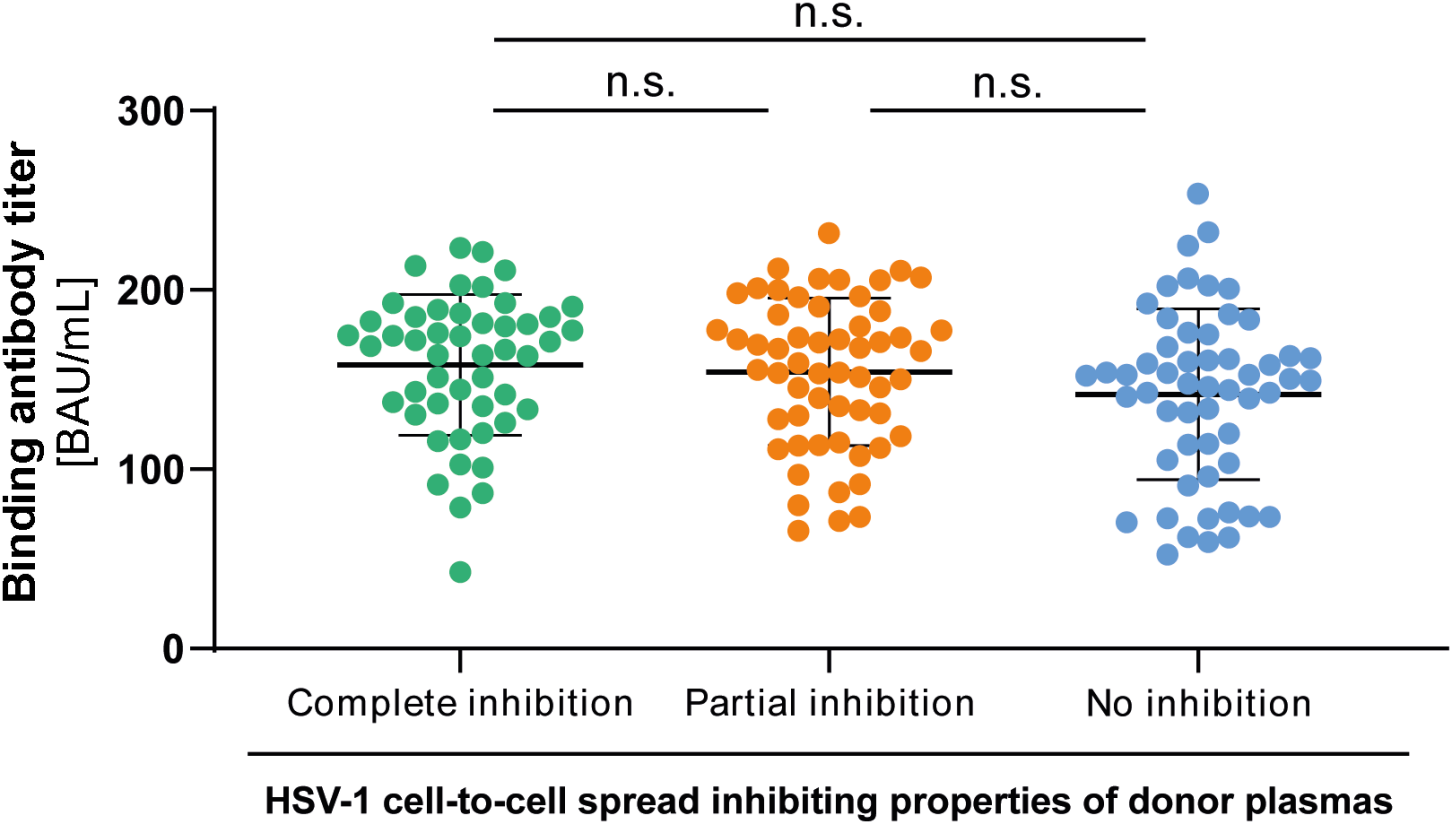
HSV-1 specific binding IgG antibody titers to HSV-1. HSV-1 specific binding antibody titers of 158 HSV-1 plasmas from blood donors that participated the retrospective survey regarding HSV-reactivations were determined by an Anti-HSV-1-IgG ELISA. The median optical density (OD450) was measured and binding antibody titers were calculated from the standard curve. Data sets were statistically analyzed using the One-way ANOVA followed by the Dunn’s multiple comparison post-hoc test. Non-significant changes (p > 0.05) are labeled as “n.s.”.

To investigate whether there may be a difference in the quantity of HSV-1 specific antibodies between the three groups, we tested all plasma samples (n = 158, complete inhibition group n = 47, partial inhibition group n = 58, and the no inhibition group n = 53) with an Anti-HSV-IgG ELISA. Plasma samples were tested at a 1:100 dilution. Corresponding binding antibody unit (BAU) concentrations were determined by standard curve plotting from median OD_450_ values. There was no significant difference in the binding antibody titers between the three groups (**Figure 5**), indicating that the quality rather than the quantity of binding antibodies was critical for elite cell-to-cell spread control.

### 3.5 Quantification of HSV-1 neutralizing antibody titers

**Figure 6:**
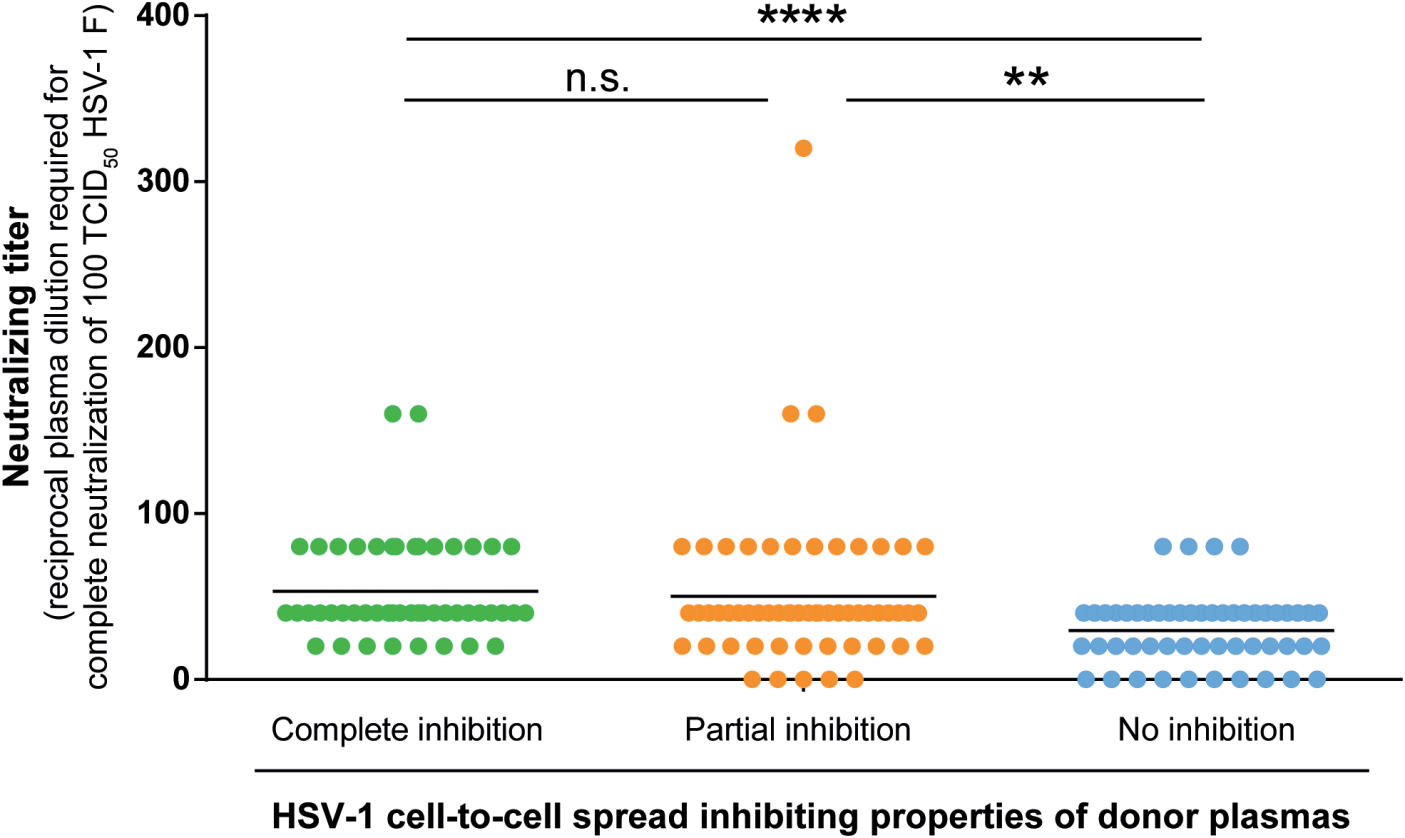
HSV-1 neutralizing antibody titers. Neutralizing antibody titers of 158 plasma samples (complete inhibition group n = 47, partial inhibition group n = 58, and the no inhibition group n = 53) were determined with a cell culture-based neutralization assay. Serial dilutions of the respective plasma samples (1:20 to 1:2560) were pre-incubated with 100 TCID50 HSV-1 F for one hour and subsequently added to confluent Vero cells in 96-well microtiter plates. After 48 h of incubation, the cytopathic effect was analyzed and the respective neutralization titers were determined. Data sets were statistically analyzed using the One-way ANOVA followed by the Dunn’s multiple comparison post-hoc test. Significant changes (**p < 0.01, ****p < 0.0001) are indicated by asterisks and non-significant changes (p > 0.05) are labeled as “n.s.”.

Next, we determined the neutralizing antibody titers of the donor plasmas against HSV-1 F in the three groups. Neutralizing antibody titers were determined with a cell culture-based neutralization assay. Serial dilutions of the respective plasma samples (1:20 to 1:2560) were pre-incubated with 100 TCID_50_ HSV-1 F for one hour and subsequently added to Vero cells. After 48 h of incubation, the cytopathic effect was analyzed, and the respective neutralization titers were determined. Plasmas that showed complete or partial inhibition of the cell-to-cell spread contained significantly higher levels of neutralizing antibodies than plasmas that could not inhibit the cell-to-cell spread (p < 0.0001; **Figure 6**). Interestingly, there was no significant difference in neutralizing titers between the complete and partial inhibition group (**Figure 6**), despite the significant difference in the frequency of reactivations between the complete inhibition group and partial inhibition group (p = 0.0009; **Figure 4**).

These results indicate that the significantly lower HSV-reactivation likelihood observed for the complete inhibition group may rather correlate with the presence of cell-to-cell spread inhibiting antibodies than with neutralizing antibodies.

### 3.6 Evaluation of cell-to-cell spread inhibition of HSV-1 F by donor plasmas

**Figure 7:**
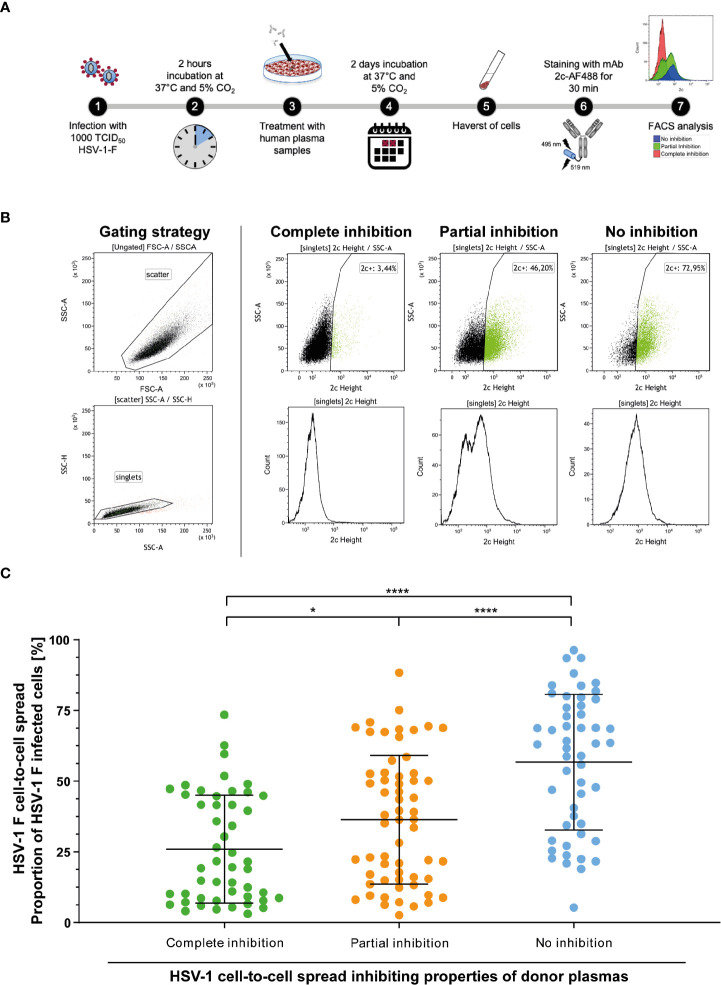
Assessment of the HSV-1 F cell-to-cell spread inhibition capacity of plasma samples against HSV-1. Plasma samples from blood donors who participated the retrospective survey (n = 158, complete inhibition group n = 47, partial inhibition group n = 58, and the no inhibition group n = 53) were re-tested for the inhibition of HSV-1 F cell-to-cell spread. **(A)** Vero cells were infected with 1000 TCID50 HSV-1 F for two hours and subsequently overlaid with plasma samples at a 40-fold dilution in cell culture medium. After two days of incubation, the cells were harvested and stained with a gB-specific, Alexa Flour 488 labeled monoclonal antibody 2c for FACS analysis. **(B)** Gaiting strategy and representative plots from cell cultures treated with plasmas that showed complete, partial or no inhibition of the HSV-1 cell-to-cell spread in the initial screening (**Figure 2**). **(C)** Percentages of HSV-1 F positive Vero cells after treatment of HSV-1 F-infected cell cultures with donor plasmas from the indicated groups. The middle horizontal bars represent the mean values and the upper and lower bars the standard deviation of the mean (SD). Significant changes were statistically analyzed using the One-way ANOVA and indicated by asterisks (*p < 0.05, ****p < 0.0001).

Initially, “elite donors” whose plasmas contain cell-to-cell spread inhibiting antibodies were identified by a newly developed high throughput HSV-1 ΔgE GFP reporter virus-based assay as described above (**Figure 1**). The glycoprotein E of herpes simplex viruses was described to facilitate the cell-to-cell spread of the virus (9). Thus, a reporter virus lacking gE may have an affected capacity of spreading *via* the cell-to-cell spread (26). The HSV-1 ΔgE GFP was used for high throughput screening because it may allow detecting of even small amounts of cell-to-cell spread inhibiting antibodies from human plasma. To verify that plasmas from previously identified “elite donors” also block the cell-to-cell spread of a HSV-1 wild type virus, we re-tested the plasmas of the participants of the retrospective survey for cell-to-cell spread inhibition of HSV-1 F (n = 158, complete inhibition group n = 47, partial inhibition group n = 58, and the no inhibition group n = 53). Inspired by a flow cytometry-based approach that was previously described for investigation of the Hepatitis C virus cell-to-cell spread (27), we adapted the assay to quantify the cell-to-cell spread inhibiting properties of HSV-1 specific antibodies (**Figure 7A**). After two days of incubation, the cells were harvested and stained with a HSV-1 gB-specific, fluorescent antibody. The proportion of HSV-1 infected cells was measured by flow cytometry (**Figure 7B**). The percentage of HSV-1 infected cells was inversely proportional to the cell-to-cell spread inhibiting properties of the plasma samples (**Figure 7B**). Overall, plasmas that completely blocked the cell-to-cell spread of HSV 1 ΔgE GFP reporter virus (**Figure 2**) also showed inhibitory properties against HSV-1 F. Notably, plasmas previously identified as completely inhibitory (**Figure 2**) inhibited the cell-to-cell spread of HSV-1 F to significantly higher degrees than plasma that previously showed partial or no inhibition (**Figure 7C**). These results provide evidence that plasmas from “elite donors” indeed contain antibodies that inhibit the cell-to-cell spread of HSV-1 (**Figures 2**–**7**).

### 3.7 Correlation analysis between HSV-1 binding, neutralizing, and cell-to-cell spread inhibition

**Figure 8:**
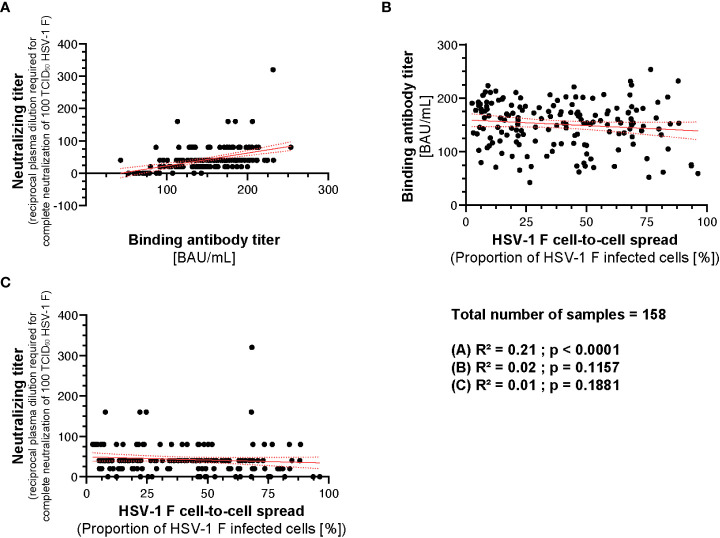
Correlation between HSV-1 IgG ELISA, neutralizing antibody titers and cell-to-cell spread inhibition. A correlation analysis was performed to investigate potential correlation between HSV-1 binding IgG titers, neutralizing titers, and cell-to-cell spread inhibition of 158 plasmas from the retrospective survey cohort was performed. **(A)** The correlation between the HSV-1 IgG ELISA binding antibody titers and neutralizing antibody titers, **(B)** HSV-1 IgG ELISA binding antibody titers and the level of cell-to-cell spread inhibition and **(C)** neutralizing antibody titers and the level of cell-to-cell spread inhibition was calculated using Spearman’s rank correlation test.

To analyze whether there is a correlation between the binding, neutralizing capacity, and cell-to-cell spread inhibition mediated by donor plasmas, we performed a correlation analysis. There was a weak correlation between the neutralizing antibody titers and binding antibody titers (R^2^ = 0.21; p < 0.0001; **Figure 8A**). However, there was no correlation between binding antibody titers and HSV-1 F cell-to-cell spread inhibition (R^2^ = 0.02; p = 0.1157; **Figure 8B**) or neutralizing antibody titers and HSV-1 F cell-to-cell spread inhibition (R^2^ = 0.01; p = 0.1881; **Figure 8C**), suggesting that cell-to-cell spread inhibiting antibodies have special quality features.

Taken together, the capability of inhibiting HSV-1 cell-to-cell spread correlates with protection from HSV reactivations. However, it is independent from its binding or neutralizing capacity.

## 4 Discussion

In the present study, we investigated whether humans are able to produce antibodies that effectively block the HSV-1 cell-to-cell spread upon natural infection. We demonstrated that humans are principally able to produce such cell-to-cell spread inhibiting antibodies against HSV-1.

In our large cohort of 2,496 blood donors, we identified a small proportion of 128 (5.1%) that had a sufficiently high antibody-concentration to block the cell-to-cell spread of HSV-1 in cell culture (elite responder). Most importantly, these individuals reported a significantly lower frequency of symptomatic HSV reactivations compared to people with lower or no detectable cell-to-cell spread inhibiting antibodies.

Interestingly, 42.5% of the plasmas showed a partial inhibition of the cell-to-cell spread, indicating that there might be cell-to-cell spread inhibiting antibodies in the plasmas, but at lower concentrations. The average concentration of antibodies in human sera/plasma was described with 11 mg/ml (28). Moreover, we showed that the average special anti-HSV-1 level did not differ between the three subgroups. In the present study, we tested the plasma samples at a 1:40 dilution, which corresponds to an average IgG concentration of 0.25 mg/mL (29). At least in 5% of individuals whose plasmas showed complete cell-to-cell spread inhibition in our high-throughput, this concentration was sufficient to prevent reinfections.

We found that there was no correlation between neutralizing antibody titers and the frequency of HSV reactivation. Despite similar binding and neutralizing antibody titers, people who had cell-to-cell spread inhibiting antibody concentrations in plasma reported significantly fewer rate of HSV-reactivations than people with insufficient concentrations of such antibodies did. These data provide evidence for the unique protective role of cell-to-cell spread inhibiting antibodies in HSV infection. These data support prior findings. Neutralizing antibodies, which are not necessarily inhibiting the cell-to-cell spread, contributed to protect from a severe course of disease (30). The presence of HSV-specific antibodies in HSV-infected mothers has been suggested to decrease the risk of acquisition of HSV-2 by newborns (31, 32). Similar findings were reported in mice. Maternal antibodies were shown to access neural tissues of the fetus or neonate, thereby protecting neonatal mice against HSV-1 neurological infection and death (32). Notably, in animal studies neutralizing antibodies blocking virus entry and cell-to-cell spread were superior to normal neutralizing antibodies that did not inhibit the cell-associated viral spread (20). These data are in line with our here presented findings.

HSV-1 is able to overcome physical barriers and escape from neutralizing antibodies by using the cell-to-cell spread (15). This mechanism is facilitated by the viral glycoproteins gD, gB, and gH/gL, which are responsible for virus entry and cell-to-cell spread (33). Previous studies described the characteristics of certain monoclonal antibodies in mice against these viral glycoproteins (15, 34, 35). It is reasonable to assume that the cell-to-cell spread inhibiting antibodies contained in plasmas of “elite donors” tested in this study target one of these antigens or even more as part of the gB, gD, gH/gL fusion machinery complex. Further follow-up studies are necessary to isolate monoclonal antibodies from the identified elite donors and to evaluate their functionality and target antigens.

In conclusion, by using a high-throughput HSV-1-ΔgE-GFP reporter assay, we have demonstrated that HSV-1-infected humans are able to produce cell-to-cell spread inhibiting antibodies. The impact of anti-gE antibodies on cell-to-cell spread was not evaluated, since we used a gE lacking HSV-1-ΔgE-GFP reporter virus for the initial high-throughput screening. Nevertheless, we were able to show that the presence of cell-to-cell spread inhibiting antibodies directly correlates with a significantly lower frequency of HSV reactivations, representing a correlate of protection. Plasmas of these individuals may be used for passive immunization strategies. Isolation of the cell-to-cell spread inhibiting antibodies may contribute to develop novel antibody-based interventions for prophylactic and therapeutic use. Moreover, characterizing of epitopes recognized by these antibodies may contribute to optimize the target antigens for novel vaccine approaches. In summary, we showed for the first time that (i) about five percent of HSV-1 seropositive blood donors (elite responder) are able to produce HSV-1 cell-to-cell spread inhibiting antibodies and (ii) that the presence of these antibodies correlates with a significantly lower risk of HSV-reactivation.

## Data availability statement

The raw data supporting the conclusions of this article will be made available by the authors, without undue reservation.

## Ethics statement

The study was performed in accordance with The Code of Ethics of the World Medical Association (Declaration of Helsinki) and was approved by the ethical committee of the University of Ulm and the University Hospital Essen (15-6360-BO). The patients/participants provided their written informed consent to participate in this study.

## Author contributions

MA, LS, SW, RD, TT, LB, MD and UA performed the experiments. MA, LS, SW, RD, MD, UA, LT, BG, MR, UD, OW, MT, CH and AK analyzed the data. MA and KR conducted the survey. RL, MR and AK planned the study. SW, LS, MA, CH and AK wrote the manuscript. All authors contributed to the article and approved the submitted version.
