# Supplementary figures and images for: Cell-to-cell spread inhibiting antibodies constitute a correlate of protection against herpes simplex virus type 1 reactivations: A retrospective study
Source: Front Immunol. 2023 Mar 16;14:1143870. doi: 10.3389/fimmu.2023.1143870 (PMC10061111; doi:10.3389/fimmu.2023.1143870)

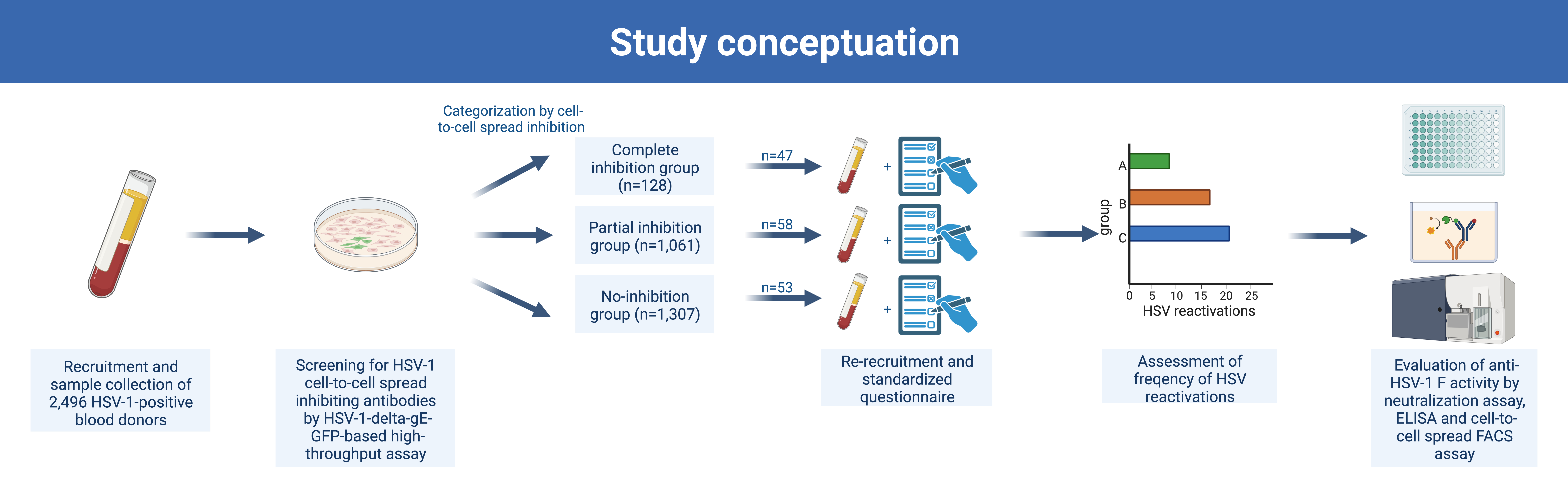

Supplement: Supplementary file 2 [file Image_1.jpeg]
